# A Two-Step Gas Chromatography-Tandem Mass Spectrometry Method for Measurement of Multiple Environmental Pollutants in Human Plasma

**DOI:** 10.1007/s11356-020-10702-6

**Published:** 2020-09-10

**Authors:** Caitlin L. Johnson, Elisa Jazan, Sek Won Kong, Kurt D. Pennell

**Affiliations:** a Department of Civil and Environmental Engineering, Tufts University, Medford, MA 02155, United States;; b Computational Health Informatics Program, Boston Children’s Hospital, Boston, MA 02115, United States;; c Department of Pediatrics, Harvard Medical School, Boston, MA 02115, United States;; d School of Engineering, Brown University, Providence, RI 02912, United States

**Keywords:** gas chromatography-tandem mass spectrometry, GC-MS/MS, chemical mixture, biomonitoring, pesticides, persistent pollutants, environmental toxicants, polychlorinated biphenyls, p,p’-dichlorodiphenyltrichloroethane, human plasma

## Abstract

Individuals are exposed to a wide variety of chemicals over their lifetime, yet current understanding of mixture toxicology is still limited. We present a two-step analytical method using a gas chromatograph-triple quadrupole mass spectrometer that requires less than 1 mL of sample. The method is applied to 183 plasma samples from a study population of children with autism spectrum disorder, their parents, and unrelated neurotypical children. We selected 156 environmental chemical compounds and ruled out chemicals with detection rates less than 20% of our study cohort (n = 61), as well as ones not amenable to the selected extraction and analytical methods (n = 34). The targeted method then focused on remaining chemicals (n = 61) plus 8 additional polychlorinated biphenyls (PCBs). Persistent pollutants, such as p,p’-dichlorodiphenyldichloroethylene (p,p’-DDE) and PCB congeners 118 and 180, were detected at high frequencies and several previously unreported chemicals, including 2,4,6-trichlorophenol, isosafrole, and hexachlorobutadiene, were frequently detected in our study cohort. This work highlights the benefits of employing a multi-step analytical method in exposure studies and demonstrates the efficacy of such methods for reporting novel information on previously unstudied pollutant exposures.

## Introduction

The assessment of risks from exposure to chemical mixtures is an important research and regulatory priority (Meek et al. 2011; Carlin et al. 2013; Kortenkamp and Faust 2018). Humans are exposed to a large number of chemicals over their lifetime, exposures which are often low-level and transient but which may nevertheless represent significant contributions to health risk (Patel et al. 2010; Nachman 2011). Despite this, the majority of exposure studies assess a limited number of environmental pollutants (Kortenkamp and Faust 2018), resulting in a lack of research data supporting the risk assessment of environmental chemical mixtures (Srivastava et al. 2017; Rotter et al. 2018).

To capture the breadth of everyday pollutant exposures, the number of chemicals measured in a single analytical method must be increased (Wild 2012). Increasing the scope of multi-pollutant analytical methods is a technical challenge that is being addressed in a variety of ways, most often through analysis of human plasma or urine using gas chromatography paired with mass spectrometry (GC-MS) and or liquid chromatography coupled with mass spectrometry (LC-MS) (Rappaport et al. 2014; Jones 2016; Weidt et al. 2016; Pan et al. 2019).

In the context of exposure assessment, analytical methods are typically classified into two categories: ‘untargeted’ and ‘targeted’ (Cajka and Fiehn 2016; Gorrochategui et al. 2016; Dennis et al. 2017). The difference between the two methods lies in whether specific chemical analytes are chosen, or ‘targeted,’ in advance during analytical method development and implementation. Untargeted methods make use of high-resolution mass spectrometry (HRMS) operated in full scan mode to detect features that are uniquely identified by mass-to-charge ratio (*m/z*) and retention time. When coupled with ultra-high-performance liquid chromatography (uHPLC), HRMS provides high resolution and high sensitivity over a *m/z* range of 50 to 6,000 with an accuracy (Go et al. 2015) of 200.0000 ± 0.0002. The mass spectral data from such platforms are subsequently mined using spectral deconvolution algorithms and chemicals are - identified based on their retention time and *m/z* (Uppal et al. 2016).

Untargeted methods are capable of detecting large numbers of chemicals in a single run, with some uHPLC-HRMS methods measuring hundreds of thousands of chemical species (Jones 2016; Uppal et al. 2016). However, there are significant trade-offs when using untargeted high-resolution methods, the main one being decreased quality and certainty of data. Specifically, detection of low-abundance environmental pollutants in biological matrices using untargeted methods is difficult and chemical identification (e.g. annotation of mass features) remains challenging (Cajka and Fiehn 2016). Quantification typically requires either a large number of internal standards or a separate/additional targeted analysis using external standards (Go et al. 2015; Savolainen et al. 2016; Gertsman and Barshop 2018). In addition, HRMS coupled with GC has only recently become commercially available (Peterson et al. 2014).

For targeted methods, chemicals of interest are chosen *a priori*, allowing the analytical method to be optimized for measurement of those specific chemicals. For the targeted analysis of lipid-soluble chemicals, gas chromatography-mass spectrometry (GC-MS) is widely used to measure multiple chemicals simultaneously and offers excellent separation and low detection limits (Barr and Needham 2002; He et al. 2015; Stubleski et al. 2018). Unlike untargeted methods, GC-MS targeted methods can detect low-abundance chemicals and reliably identify and quantify chemicals using standard calibration and quality control procedures. However, designing and implementing a calibrated and quality-controlled GC-MS method for a large number (e.g., n > 50) of chemicals requires a considerable amount of time and effort. Methods targeting an even greater number (e.g., n > 100) of chemicals often forgo some traditional measures of quality control and employ a limited number of calibration standards to save time and cost (European Commission Directorate Genreal for Health and Food Safety, 2018). Pooling of multiple plasma samples is also used in some studies (including the National Health and Nutrition Examination Survey, or NHANES) to reduce analytical expense and reduce non-detects (Bates et al. 2005; Caudill et al. 2007).

For targeted multipollutant analytical methods applied to human plasma samples, there is a risk that many of the chemicals in a multipollutant method will not be detected in the study population at sufficient frequency for robust statistical analysis. For example, Younglai et al. (Younglai et al. 2002) detected only 6 of 71 targeted chemicals in > 50% (n participants = 21) of serum samples. Thomas et al. (Thomas et al. 2006) detected only 25 of 75 organohalogen chemicals in > 50% of study participants (n participants = 154). This is a natural consequence of the low-level multi-pollutant exposure paradigm where detection of exposure to individual chemicals is sparse.

**Fig 1:**
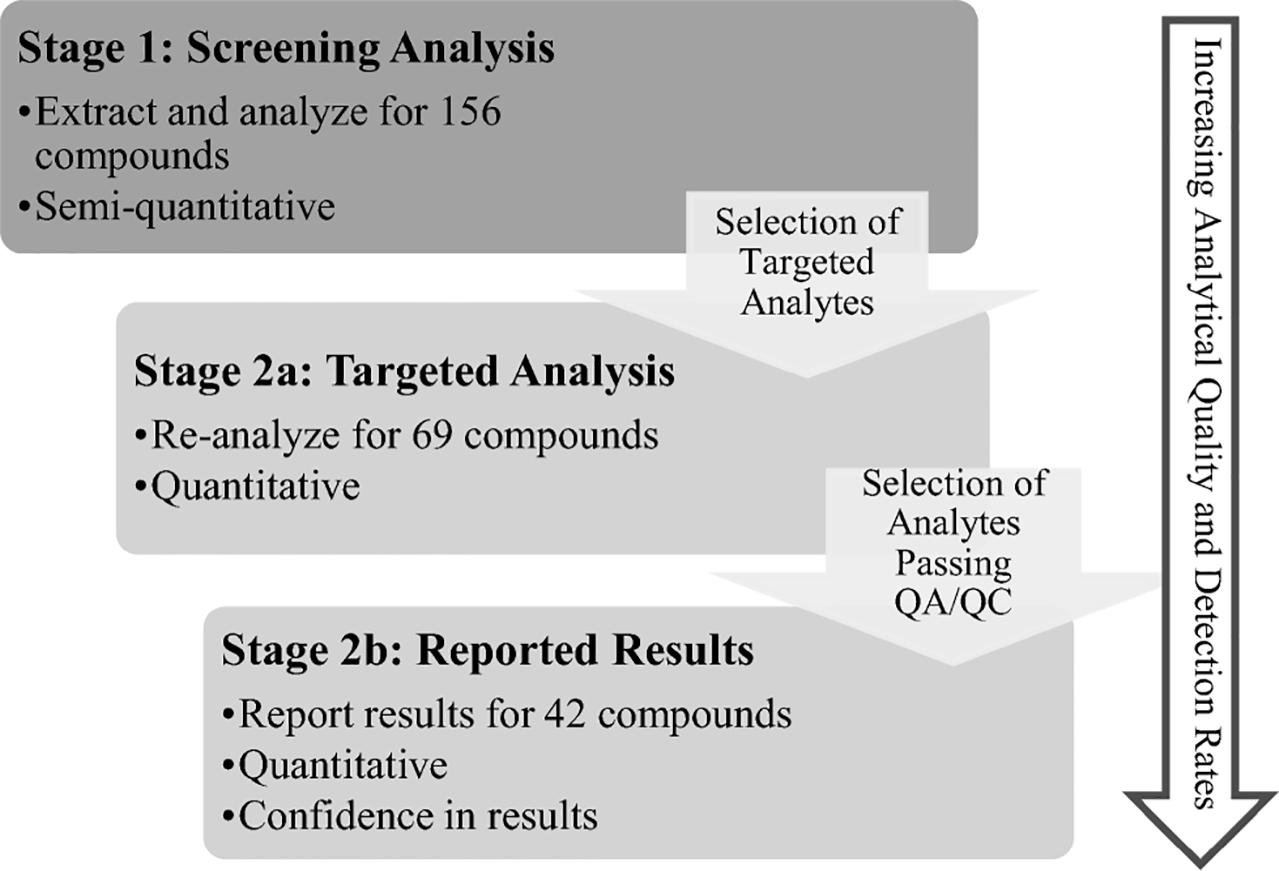
Flow chart of the analytical process from initial screening to reporting results

To address these issues, we developed a two-step analytical method (Figure 1) consisting of a semi-quantitative screening step (156 chemicals) followed by targeted analysis of a subset of chemicals meeting the initial screening criteria (69 chemicals). To demonstrate a real-world application of the method, we used the method on plasma samples (n = 183) of children with autism spectrum disorder (ASD), their parents, and unrelated neurotypical children from an existing cohort at the Boston Children’s Hospital (Kong et al. 2012). We compared the resultant plasma concentrations of 42 chemicals between adults and children (regardless of ASD diagnosis) and between children with ASD (probands) and neurotypical children (controls). Adult plasma concentrations were also compared to United States median pollutant exposures from NHANES. Due to the wide scope of the method, we also reported plasma concentrations of five compounds which are rarely or never measured in human plasma.

## Materials and Methods

### Samples

Plasma samples from 183 individuals, including children with ASD (i.e., probands), their parents, and unrelated neurotypical children were obtained from the Boston Children’s Hospital Biobank (Table 1). Criteria for inclusion for the probands was age >24 months and a clinical diagnosis of ASD by Diagnostic and Statistical Manual of Mental Disorders, Fourth Edition, Text Revision (DSM-IV-TR) criteria as previously described (Kong et al. 2012). Family groupings included 37 mother/father pairs, 33 father/proband pairs, and 32 mother/proband pairs. Although the purpose of this study is method development, we used samples from a cohort studying ASD to demonstrate the utility of this method for assessing exposures in an epidemiological context.

### Reagents and Standards

Hexane (98.5% purity), acetone (99.5% purity), methyl tert-butyl ether (MTBE; >99% purity), dichloromethane (DCM; 99.9% purity) and anhydrous sodium sulfate (99% purity) were purchased from Thermo Fisher Scientific (Waltham, MA). Florisil solid-phase extraction (SPE) tubes were purchased from Thermo Fisher Scientific and UCT, Inc. (Bristol, PA). Nitrogen and Helium gases (99.999% purity) were purchased from Airgas (Billerica, MA).

A multi-standard was constructed by combining standard mixes of common environmental pollutants. A full list of the names of the chemicals used, their chemical abstract service (CAS) numbers, and their commonly used abbreviations can be found in Online Resource 1. The following standard mixtures were purchased from Accustandard (New Haven, CT): CLP Toxic Substance Mix (CLP-HC-SV-MIX4), AccuGrand 8270 Semi-Volatile Standard (M-8270-AG01-ASL), AccuGrand 8270 Semi-Volatile Standard (M-8270-AG02-ASL), Organonitrogen Pesticides Mix (M-633), Halogenated Volatiles Mix (M-8010B), Aromatic Volatiles Mix (M-8021B-AV), Method 525.2 Organochlorine Pesticides (M-525.2-CP-ASL), polybrominated diphenyl ethers (PBDE) Congeners of Primary Interest Calibration Mix (BDE-CM), polychlorinated biphenyl (PCB) Congeners Mix 2 (AE-00041), Pesticide Mix 1 (AE-00010), Pesticide Mix 18 (AE-00028), Furan Mix (M-8280B), Pesticide/Herbicide Mix (M-551.1C), and WHO/NIST/NOAA Congener List (C-WNN). Individual standards for 2-ethylhexyl 2,3,4,5-tetrabromobenzoate, pentabromoethylbenzene, tris(2-chloroethyl)phosphate (TCEP), 2,2’,3,4,4’,5,5’-heptabromodiphenyl ether (BDE 180), di(2-ethylhexyl)tetrabromophthalate (TBPH), and PCB congeners 65 and 166 were also purchased from Accustandard. SV Calibration Mix #5/610 polycyclic aromatic hydrocarbon (PAH) Mix (Product Number: 31011) was purchased from Restek (Bellefonte, PA). From these standard mixtures, a 258-component multi-standard was constructed in hexane. Microsoft Access (Microsoft Office Professional Plus 2010; Version 14.0.7232.5000) was used to track individual chemical properties and compute the final concentration of each chemical in the multi-standard.

### Extraction Procedure

The extraction procedure was based on methods by Hatcher-Martin et al. (Hatcher-Martin et al. 2012). Briefly, frozen plasma samples (225–450 μL) were thawed. The thawed plasma was added to 5 mL of a 1:1 hexane/acetone mixture in an amber glass vial, along with 10 μL of 70 ngm/L PCBs 65 and 166 as internal recovery surrogates. Extracts were vortexed and allowed to equilibrate at room temperature overnight (ca. 12 hours). The sample extract was then transferred, along with two 3 mL rinses, to a 5 g Florisil SPE cartridge containing 1 g anhydrous sodium sulfate that had been preconditioned with 10 mL of MTBE. The SPE cartridges were eluted twice with 5 mL of MTBE and once with 5 mL of dichloromethane. The eluent was then reduced to ca. 1 mL by heating to 40°C under a gentle stream of ultra-high-purity nitrogen gas. Care was taken to never evaporate samples to dryness to avoid loss of semi-volatile chemicals. Samples were transferred to clear autosampler vials along with two 0.5 mL hexane rinses using glass Pasteur pipettes and evaporated to ca. 0.1 mL at 45°C under a gentle stream of ultra-high-purity nitrogen. Samples were then transferred along with two 50 μL hexane rinses to pre-weighed amber autosampler vials with glass high-recovery inserts. Vials were capped, weighed, and stored at 4°C until analysis. The average storage time was 2 months.

### Instrumental Analysis—Step 1: Screening Method

The instrument used for analysis was an Agilent 7010 Gas Chromatograph-Triple Quadrupole Mass Spectrometer (GC-QQQ, Agilent Technologies) equipped with a Gerstel Cooled Injection System (CIS; GERSTEL Inc.). Separation was achieved with a 30-m long DB-5MS Ultra Inert column (250 μm phase thickness, 0.25 mm inner diameter; Agilent Technologies) using helium as the carrier gas at a flow rate of 1 mL/min. The inlet was held initially at a temperature of 70°C and then increased to 300°C at a rate of 12°C/second and held at that temperature for 10 minutes. The oven temperature was initially set at 70°C, held for 2 minutes, increased to 150°C at 20°C/min, increased to 200°C at 3°C/min, increased to 310°C at 8°C/min, and then held for 7 minutes.

Although the multi-standard contained 258 chemicals, preliminary investigations with standard mixes showed that only 156 chemicals were feasible for GC-MS measurement using the extraction and analytical method described herein. The mass spectrometer was operated in multiple reaction monitoring (MRM) mode with two transitions monitored for each of these 156 chemicals (names and MRM transitions listed in Online Resource 1). Transitions and collision energies were optimized using Agilent’s MRM Acquisition Optimization Tools (Version B.07.00). A dwell time of 10 ms was used for each chemical and MRM transitions were monitored over limited time segments which were optimized to keep the cycle time in each time segment below 600 ms. MassHunter Quantitative Analysis for QQQ (Version B.07.01, Agilent Technologies) was used to integrate and quantitate m/z features.

#### Screening Method Quality Control

Applicability of the GC-MS method was assessed by the linearity of the calibration curve and performance of the quality control samples. A six-point calibration curve over two orders of magnitude (< 0.01 ng/mL to > 10 ng/mL for most chemicals) was attempted for each of the 156 chemicals. To address potential background matrix effects of the serum residue on sample concentration, the initial calibration samples and all continuing calibration samples were prepared by extracting 450 μL of charcoal-stripped fetal bovine serum (FBS; Sigma Aldrich) and then adding the appropriate amount of a concentrated multi-standard. Calibration curves were considered acceptable if at least three calibration levels were detected, and the calibration curve had an R^2^ of > 0.8. Chemicals not meeting these criteria were excluded from the targeted method.

Extraction method recovery was assessed using spiked and unspiked donor serum. Donor serum samples consisting of serum from a single human donor (Lee BioSolutions, Inc.) were extracted and analyzed alongside the samples either unspiked (n = 2) or spiked (n = 6) with 30 μL of a stock solution to a final concentration of 1.2–180 ng/mL (ca. 12 ng/mL for most chemicals). Percent recovery was calculated as the difference in the average measured concentrations of donor serum spikes and donor serum samples divided by the concentration added. Since it was possible that recovery would improve in the targeted method, quality control samples for the screening method were evaluated holistically alongside other screening criteria. Quality control sample results alone were not used to exclude a chemical from the targeted method unless they indicated very low (< 50%) recovery. No precision cutoffs were used for the screening method, other than the R^2^ cutoff of 0.8.

#### Screening Method Detection Rates

For the screening method, traditional limits of detection were not calculated. Instead, chromatogram peaks were considered detects if they met the following four criteria:

- Retention time within ± 0.1 minutes of the calibration standard’s retention time
- Peak area above that of the most recent instrumental blank
- Peak shape similar to that of the calibration
- Qualifier/quantifier ratio (if applicable) within ± 20% of the calibration standards

Detection of a chemical in < 20% of samples resulted in that chemical being excluded from the targeted method.

The analytical background was assessed using an instrumental detection limit (IDL) calculated for a subsample of 31 compounds using ten replicate injections of a low-concentration standard.

### Instrumental Analysis—Step 2: Targeted Method

The targeted analysis employed the same instrumental method as the screening analysis except that the number of monitored MRM transitions was lower (ca. 140 vs. 312 for the screening method). This allowed decreased cycle time (ca. 400 ms) which increased the number of data points collected over each chromatographic peak. Based on the targeted analysis and results of quality assurance samples, results were reported for 42 chemicals (listed in Table 2) after quality control procedures showed those chemicals to have good accuracy (percent error <50% on National Institute of Standards and Technology (NIST) Standard Reference Material (SRM) 1958 samples for two or more batches), low inter-sample variability (matrix spike RSD <60%), and low inter-batch variability (Kruskal-Wallis test on matrix spikes with a probability cut-off of p < 0.01).

The plasma sample extracts were analyzed in five randomly assigned batches of 40 samples + 10 quality control samples. Each sample was run in duplicate on the GC-QQQ and the concentrations calculated from the two injections were averaged to produce a final concentration. In between each batch the GC inlet liner was replaced and the entire system was baked out: the inlet and column were heated at 300°C for 45 minutes, and then the source and quadrupoles were heated at 300°C and 200°C respectively for 4 hours. The mass spectrometer was autotuned after the baking out procedure to maintain optimal performance.

#### Targeted Method Calibration and Quality Control

Matrix-matched six-point calibration curves covering four orders of magnitude (0.005 ng/mL to 10 ng/mL for most chemicals) were prepared in the same manner as the screening method. Calibration curves were fit as either an unweighted linear regression or a linear regression with weighting equivalent to 1/[sample concentration].

Several external quality control samples were extracted and analyzed alongside plasma samples. These consisted of: (1) method blanks, (2) matrix spikes, and (3) aliquots of National Institute of Standards and Technology Standard Reference Material 1958 (NIST SRM 1958).

##### Method Blanks.

Method blanks (n=3–8 per batch, 25 total) consisting of blank charcoal-stripped FBS were used to assess method contamination. Blank subtraction was used if the average method blank responses for a specific chemical/batch were >10 times higher than the lowest calibration standard used and the RSD of the method blanks for the chemical/batch were less than 50%.

##### Matrix Spikes.

Matrix spikes (n=5–8 per batch, 29 total), consisting of charcoal-stripped FBS spiked with 9 to 225 ng/mL of chemicals (concentration varied by chemical), were used to identify chemicals that exhibited high inter-sample or inter-batch variability. Inter-sample variability was assessed by computing the relative standard deviation (RSD) of the matrix spikes from all batches. Chemicals were excluded for high inter-sample variability if the relative standard deviation was greater than 60%. Chemicals were also excluded if the matrix spike values were determined to be different between batches using a non-parametric Kruskal-Wallis test with a probability cut-off of p < 0.01.

##### NIST SRM 1958.

NIST SRM 1958 samples (n=3 per batch, 15 total) were used to determine the accuracy of the analytical method for 33 chemicals. The NIST SRM 1958 reference material is freeze-dried serum which contains spiked concentrations of commonly measured PCBs, PBDEs, and chlorinated pesticides. The reported concentrations of these chemicals in the NIST SRM 1958 material are certified by analysis at NIST, the Centers for Disease Control and Prevention, and several private laboratories. Chemicals were excluded from further analysis due to poor accuracy if the absolute value of the percent error of the NIST SRM 1958 samples was greater than 50% for more than one batch.

#### Limits of Detection

For the targeted analysis of 42 chemicals, limits of detection (LOD) were estimated using low-level spiked standards (n=10). $\text{LOD}\sqrt{2}$ was substituted for values below the LOD for the purposes of statistical testing, a method which is shown to generate relatively low bias for datasets with < 50% censoring (Antweiler and Taylor 2008; Antweiler 2015).

### Statistical Analysis

Medians were not computed for chemicals with < 50% detects and chemicals with < 30% detects were not used in statistical analyses (Antweiler and Taylor 2008; Antweiler 2015). All computations and statistical tests of the results were performed using in the statistical language R (Version 3.6.1) (R Core Team 2019). We used non-parametric statistical tests due to the skewed distribution of the data and the presence of non-detects. For comparison of two groups, rank-sum tests (wilcox.test() function in R) were used and p-values were corrected using the Bonferroni adjustment by selecting an initial p-value cut-off of 0.05 and dividing it by the number of chemicals with ≥ 30% detects (n=23) for an adjusted p-value cut-off of 0.002. Spearman correlations between measured proband and parent chemical concentrations were computed using the base function cor() and were plotted using the additional packages plyr (Wickham 2011), ggplot2 (Wickham 2016), and reshape2 (Wickham 2007).

## Results and Discussion

### Screening Method

**Fig 2:**
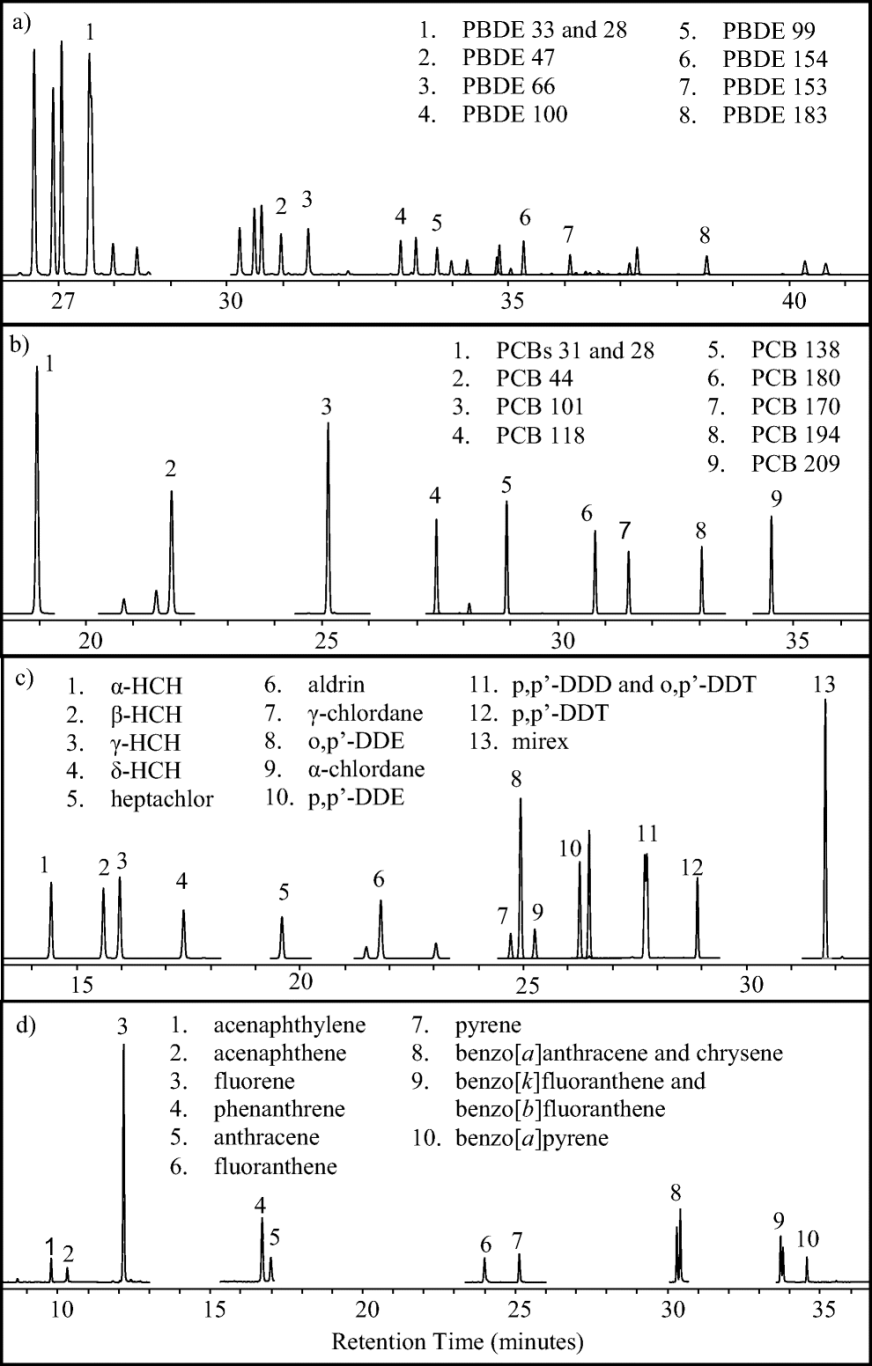
Chromatograms obtained for representative chemical classes showing area counts vs. retention time in minutes for: a) brominated flame retardants, b) polychlorinated biphenyls, c) organochlorine pesticides and their derivatives, and d) PAH). Note that, although there appear to be coeluting chemicals in chromatograms (c) and (d), these are identified by multiple reaction monitoring

**Fig 3:**
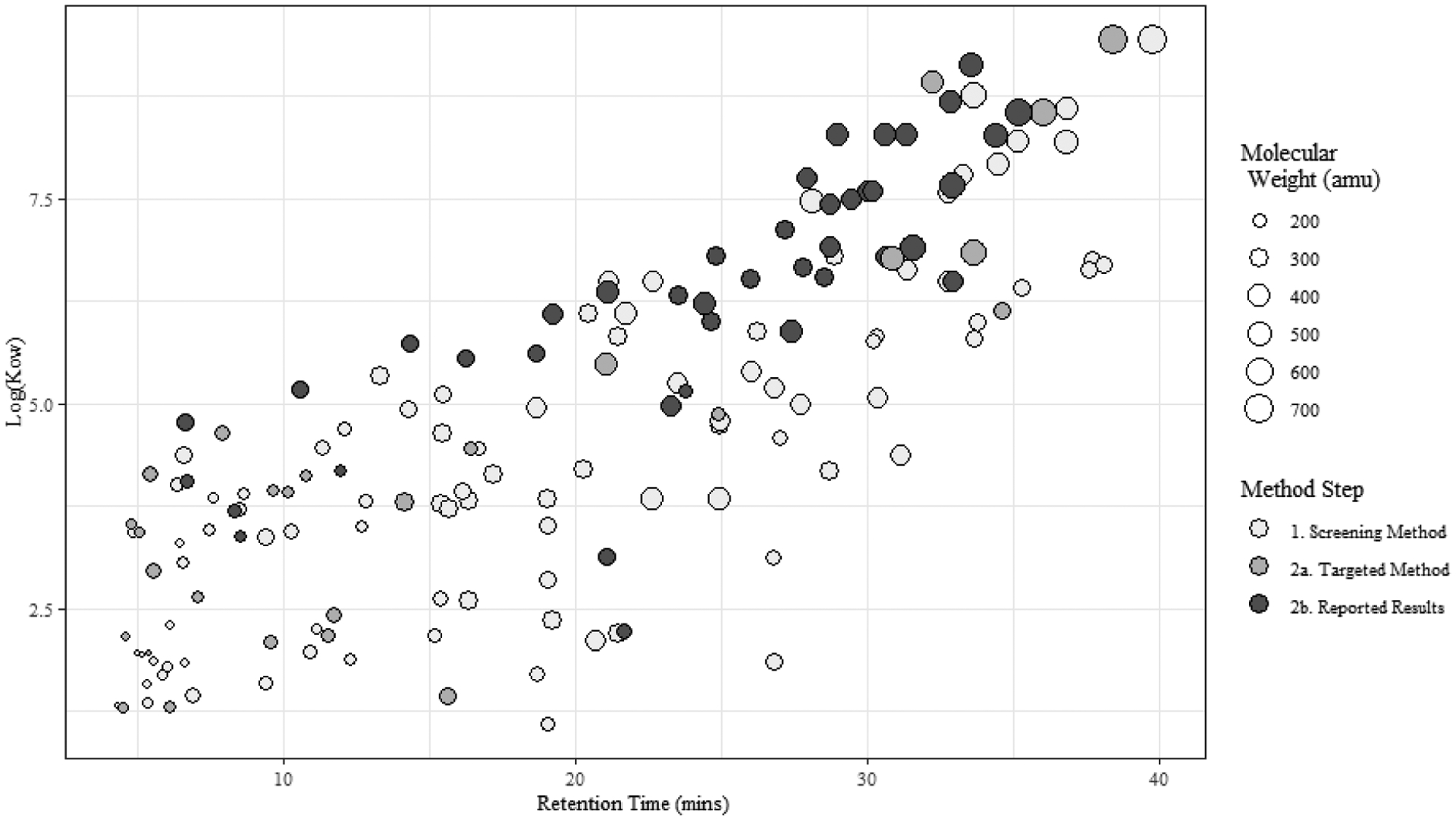
Chemicals included in each stage of the method where Log(K_ow_) is the logarithm of the octanol-water partition coefficient, and retention time is an indicator of compound volatility. Dot size indicates molecular weight of the chemical, where each dot represents a chemical that was included in the screening method. Dots are shaded to indicate whether the chemical was eliminated from study after the screening method (no shading), eliminated from study after the targeted method (grey shading), or not eliminated (i.e. final results were reported; black shading)

Using a combination of chromatography and MRM, a total of 156 chemicals were separated with sufficient resolution to allow for quantification (example chromatograms shown in Figure 2). Of the 95 chemicals that did not pass the screening, 34 did not pass the analytical quality criteria and another 61 were detected in < 20% of samples. The 61 chemicals that passed the screening were generally representative of the overall chemical selection (Figure 3) with respect to log K_ow_ (proportional to hydrophobicity) and retention time (proportional to volatility). Analysis of quality control samples showed recoveries above 50% for all the chemicals that passed the screening except for diethyl phthalate, fluorene, oxychlordane isomer, and PBDEs 28 and 99. Instrumental detection limits for the 31 chemicals tested were all below 0.1 ng/mL except for δ-HCH (Online Resource 2). Eight more PCBs of toxicological interest (congeners 66, 105, 156, 157, 167, 187, 195, and 206) were added to the targeted method for a total of 69 chemicals.

The results of the screening method, although primarily used to select chemicals for the targeted method, yielded valuable information about the chemical exposures of the study cohort. Chemicals that were detected in < 20% of samples in the screening method are likely ones for which the overall cohort has low exposure. Although this was not useful for data comparisons between cohorts, it could help guide future exposure studies within the same cohort. Conversely, chemicals that were detected in the screening method but did not produce quantitative results in the targeted method may be studied at a later point using more specific or higher sensitivity targeted methods since these chemicals are known to be detectable.

The screening method was effective in limiting the scope of the targeted method to detectable and quantifiable chemicals. From the initial group of 156 chemicals, 61 were excluded because of low detection rates despite acceptable analytical criteria, suggesting that these chemicals would not be suitable for subsequent statistical analysis regardless of analytical method. In the final results of the targeted method, a total of 25/69 (36%) chemicals from the targeted method had >50% detection rate. This detection rate is substantially higher than if a targeted method had been attempted for all 156 chemicals (25/156, or 16%) without the initial screening step (Figure 1). The detection rate was also higher than recent studies by Younglai et al. (Younglai et al. 2002) and Thomas et al. (Thomas et al. 2006), which reported 6/71 (8.5%) and 25/75 (33%) chemicals with >50% detects in human plasma samples.

### Targeted Method

Of the 69 chemicals measured using the targeted analytical method, concentrations were reported for 42 chemicals (Table 2). The combined concentration of PCBs 28 and 31 was reported as a sum due to coelution of these isomers. The average detection rate in the plasma samples was 48%, with 25 chemicals detected in >50% of samples.

Targeted method results were generally consistent with similar exposure studies. For example, p,p’-DDE was detected in almost all (99% of) samples using the targeted method, similar to other studies measuring multiple chemicals in human serum (Botella et al. 2004; Thomas et al. 2006; Vriens et al. 2019). PCB congeners 118 and 180, which were both detected in >85% of plasma samples, have been detected with high frequency in other studies as well (Thomas et al. 2006; Sethi et al. 2019; Vriens et al. 2019).

Plasma concentrations for thirteen chemicals measured in this study for adults (age 32–53 years old) were compared to national medians for 30–50-year-olds in the United States measured by the National Health and Nutrition Examination Survey (NHANES; Table 3). In general, adults in this study had a median plasma concentration similar to (between 0.5–3 times) the NHANES median value, except in the cases of PCB 66 (5.8x higher than NHANES) and PCB 206 (3.5x higher than NHANES). This is expected as the adults in the study were not known to have any exceptional chemical exposure relative to the general population. Differences in specific chemicals relative to the NHANES median may be due to variation in pollutant exposures across regions or individual variability.

Some chemicals that are not commonly measured in human serum/plasma were detected at high rates. Two PAHs, fluorene and fluoranthene, which are typically measured in human urine samples as hydroxylated adducts (Jacob and Seidel 2002; Castaño-Vinyals et al. 2004), were detected in 77% and 90% of samples respectively. 2,4,6-Trichlorophenol, also typically measured in urine (Ye et al. 2008), was detected in 82% of samples at concentrations up to 1.8 ng/mL. Isosafrole, a fragrance chemical which has, to our knowledge, never been measured in human biomatrices, was detected in 75% of samples at concentrations up to 3.41 ng/mL. Hexachlorobutadiene, another chemical not routinely measured in human plasma, was detected in 65% of samples at concentrations up to 4.30 ng/mL. The detection of these chemicals at high frequencies in plasma samples from both adults and children has several implications. For chemicals with limited information on chronic health effects in humans, such as isosafrole (European Commission, Health and Consumer Protection Directorate, 2003) and hexachlorobutadiene (Agency for Toxic Substances and Disease Registry 1994), these results suggest new directions for biospecimen collection in epidemiological studies. Researchers without access to urine specimens may still be able to measure these chemicals in their studies if plasma samples are available. The detection of PAHs and 2,4,6-trichlorophenol, which are typically measured in human urine, in the plasma samples from this study suggests that human plasma may be useful matrix for biomonitoring of these chemicals.

#### Calibration and Quality Control

Two chemicals, tris(2-Chloroethyl) phosphate (TCEP) and diethyl phthalate, could not be quantified due to high background contamination. Aside from these two chemicals, calibration R2 values were greater than 0.8 for all chemicals in all batches except for Batch 1, in which three chemicals (3-methylphenol, n-nitrosodi-n-butylamine, pentachlorobenzene) yielded calibration R2 values between 0.6–0.8, and in Batches 2 and 3, in which PBDE 47 yielded a calibration R2 of 0.7–0.8. All of these chemicals except pentachlorobenzene were later excluded based on quality control criteria (high batch and inter-sample variability in the case of 3-methylphenol and n-nitrosodi-n-butylamine; poor accuracy in the case of PBDE 47). The average calibration R2 across all chemicals and batches was 0.967 (standard deviation = 0.050).

##### Method Blanks.

Based on method blank results, blank subtraction was used for 1,3-Dichlorobenzene in Batch 4, p,p’-DDE in Batch 1, and PCB 138 in Batch 4.

##### Matrix Spikes.

Matrix spike analysis revealed nine chemicals with high inter-batch variability as indicated by the Kruskal-Wallis test on sample results and a relative standard deviation of >60% between matrix spikes from different batches. These nine chemicals (3-methylphenol, n-nitrosodi-n-butylamine, 2,6-dinitrotoluene, acenaphthene, dibenzofuran, N, N-diethyl-meta-toluamide (DEET), phenanthrene, pyrene, PCB 195, and PBDE 183) were excluded from further statistical analysis.

##### NIST SRM 1958.

Percent errors relative to NIST SRM 1958 certified concentrations were generally within ±50% (Table 4), except for five chemicals: oxychlordane isomer and PBDE congeners 47, 99, 153, and 183. These five chemicals were excluded from further statistical analysis and their concentrations are not reported here. NIST SRM results for p,p’-dichlorodiphenyltrichloroethane (DDT) in Batches 1 and 2 were not useable due to the presence of a coeluting peak with a slightly different retention time, which was observed in the NIST SRM but not in the study samples.

Thirteen chemicals from the targeted method could not be measured in Batch 5. Samples for Batch 5 were stored for several months while instrument repairs were made, so it is likely that these chemicals were too volatile to remain in storage for that long. Although the concentrations for most of these compounds were not reported due to inter-batch variability, three of these chemicals (hexachlorobutadiene, 1,2,3-trichlorobenzene, and isosafrole) were included in the following group comparison analyses and their summary concentrations are included in Table 2. Statistical tests for these three chemicals only used results for Batches 1–4.

### Group Comparisons

Median concentration values were computed for each chemical with >50% detects for adults and children (both probands and controls) separately (Table 2). For five chemicals, indicated in Table 2 with an asterisk (*), the plasma concentrations of the adults were significantly (p < 0.002) different from those of the children. Adults had higher levels of most PCBs and organochlorine pesticides than children, a finding that is consistent with existing research showing that persistent organic pollutant concentrations increase with age (Alcock et al. 2000; Nichols et al. 2007; Singh et al. 2019). Some chemicals, notably fluorene and PCBs 18 and 101, were higher in children than adults although the trend was not significant. Plasma concentrations of fluoranthene, the only other PAH measured, were also slightly (but not significantly) higher in children, suggesting that PAH plasma concentrations may be less age-related than other persistent organic pollutants. Several other studies have reported that children have high plasma concentrations of PCBs (Caspersen et al. 2016), possibly due to differences in physiology and dietary intake between children and adults (Grandjean et al. 2008). This highlights the large variation in relative PCB congener concentrations across cohorts, which are best observed by measuring multiple PCBs within a method.

No significant (p < 0.002 after Bonferroni adjustment for multiple tests) differences in individual chemical concentrations were observed between probands and controls. In addition, there were no significant differences (p < 0.002) between chemical levels in the mothers and fathers. Several previous studies of different populations have found significantly higher levels of PCBs in women compared to men (Qin et al. 2011; Porta et al. 2010; Pirard et al. 2018). This may not have been observable in the current study since all of the women in this study were mothers and thus might have breastfed an infant, an activity which decreases pollutant body burden (Thomas et al. 2006; Bjermo et al. 2013; Pirard et al. 2018).

### Family Relationships

**Fig 4:**
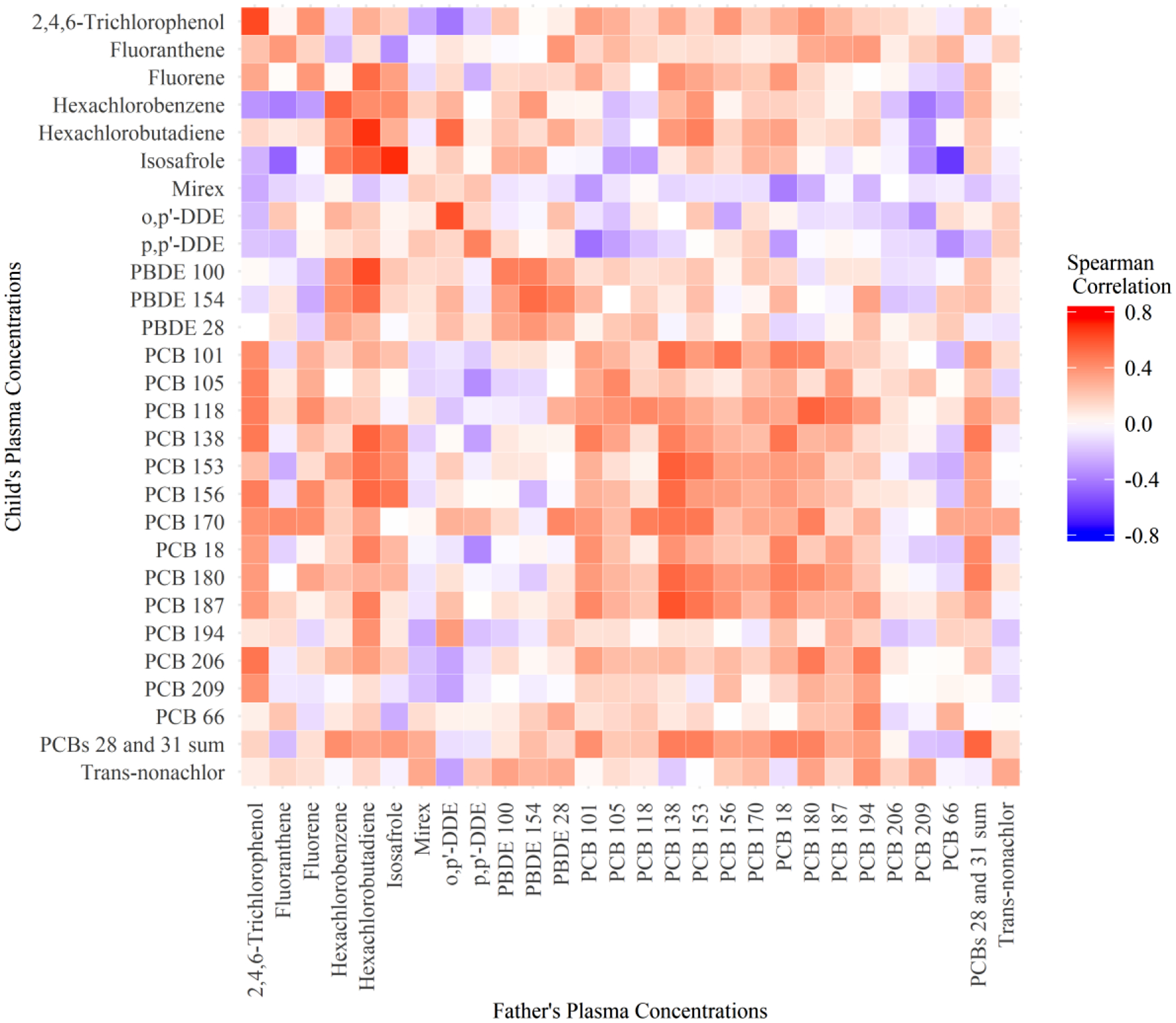
Correlation heatmap of children and their fathers’ plasma chemical concentrations. Higher correlation coefficients along the diagonal indicate pollutants which displayed similar concentrations between fathers and their children

**Fig 5:**
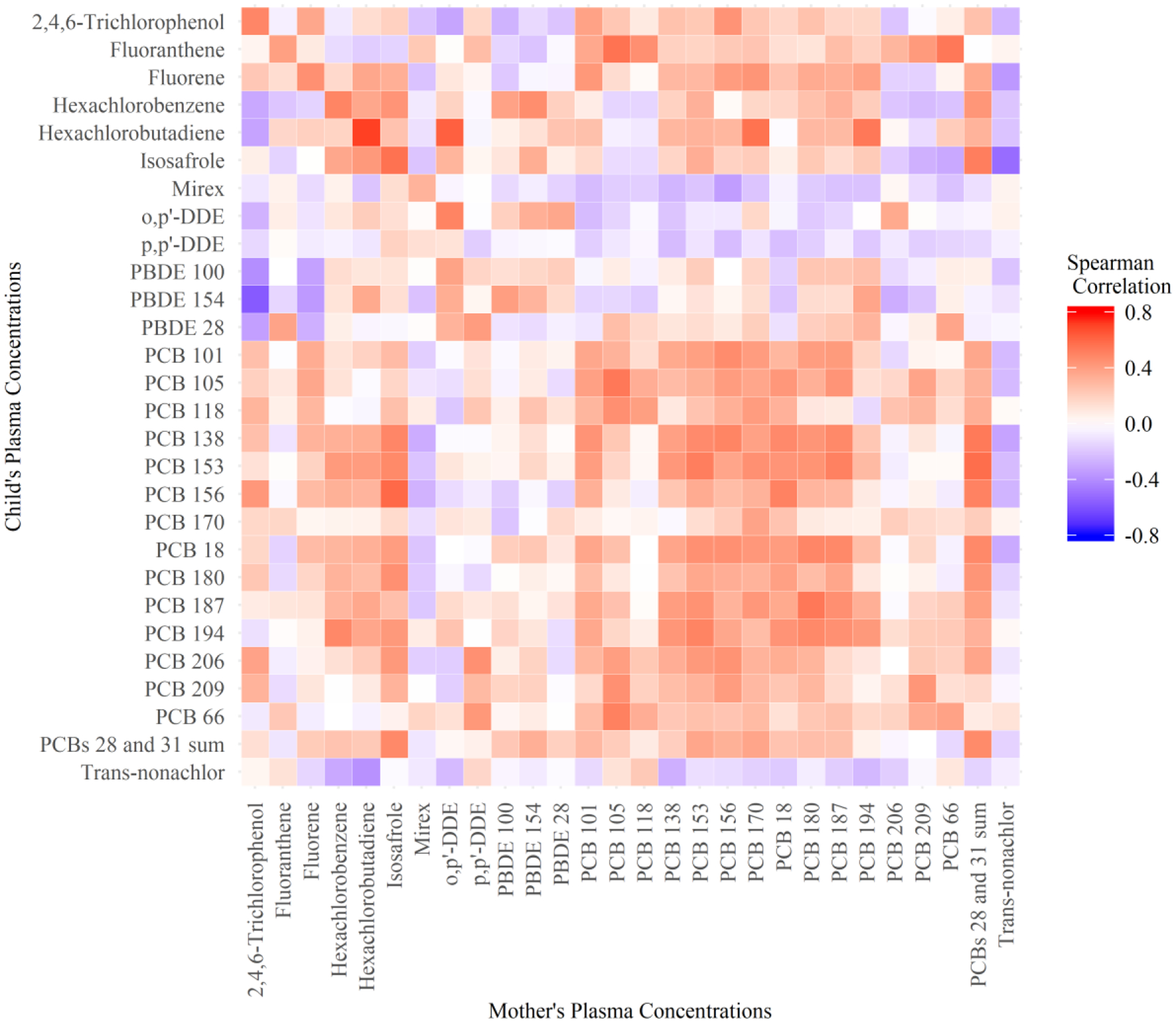
Correlation heatmap of children and their mothers’ plasma chemical concentrations. Higher correlation coefficients along the diagonal indicate pollutants which displayed similar concentrations between mothers and their children

Heatmaps displaying the correlation between the plasma concentrations measured in children diagnosed with ASD and their mothers and fathers are shown in Figures 4 and 5. Of particular note are isosafrole, hexachlorobutadiene, o,p’-DDE, and 2,4,6-trichlorophenol, which were correlated between children and their father’s plasma concentrations with a Spearman’s rho of 0.6 or higher. For children and their mothers, children’s hexachlorobutadiene plasma concentrations correlated with mothers’ hexachlorobutadiene and o,p’-DDE plasma concentrations (ρ ≥ 0.6). More generally, PCB concentrations in parents’ plasma samples were correlated with children’s PCB plasma concentrations, except for PCBs 206 and 209.

The correlations between plasma chemical concentrations of children and their parents highlights the role that shared environments might play in determining chemical exposures. Previous studies have found correlations between parents and children’s serum PBDE concentrations (Wu et al. 2015; Knudsen et al. 2017), and between maternal PCB exposure and children’s plasma PCB levels (Fängström et al. 2005; Caspersen et al. 2016). Several studies have also observed correlations between family members’ phthalate exposures (Sathyanarayana et al. 2008; Kasper-Sonnenberg et al. 2012; Koppen et al. 2019). However, the results provided here represent, to the best of our knowledge, the first evidence of shared household exposures for several chemicals including isosafrole, hexachlorobutadiene, and 2,4,6-trichlorophenol. The measurement of multiple chemicals in a population also allows for observations within chemical classes, such as the relatively low inter-family correlations of high-molecular-weight PCBs. Although these results, which are based on families with an autistic child, cannot be generalized to the average US population, they nevertheless suggest that future study of parent-child shared exposures is warranted.

## Conclusions

Due to naturally low concentrations of environmental pollutants in human plasma, it can be difficult to choose chemicals for analysis which will be present in samples at rates high enough for meaningful statistical analysis. Pre-screening of samples for detection rates provides a solution to this problem. In this paper, we present a two-step method that enabled the screening of 156 environmental pollutants using a widely available and relatively inexpensive GC-MS/MS platform. This two-step approach was applied to 183 plasma samples collected from children and adults in the north-eastern US with no known chemical exposures. Despite the expected low concentrations of pollutants in the subjects’ plasma samples, detect/non-detect information was obtained for 130 chemicals, and quantitative concentration data was collected for 42 chemicals. Due to successful pre-screening of compounds with low rates of detection in the sample population, the targeted method used here had a much higher success rate detecting chemicals than recent targeted studies of similar size.

Using these quantitative data, we were able to report the first values for human internal concentrations of isosafrole and hexachlorobutadiene. We also measured three chemicals (fluorene, fluoranthene, and 2,4,6-trichlorophenol) in plasma which are usually measured in urine, providing additional perspective on internal exposures to these compounds. Furthermore, we explored correlations between family members’ plasma samples for these and 23 other compounds, including chlorinated pesticides, PBDEs, and PCBs. This innovative use of a widely available GC-MS/MS platform allows analytical laboratories to increase the number of chemicals included in biomarker analyses without the use of high-resolution mass spectrometry instrumentation.

The targeted two-step GC-MS/MS method described herein is not without limitations. The number of chemicals was limited by available standards and the logistics of combining standards to generate independent calibration curves. This method requires more time than a single targeted instrument run; for each sample there are two instrumental runs, two data analyses, and a selection process to determine chemicals to be included in the targeted method. These limitations can be overcome by newer platforms such as the GC-Orbitrap-MS, which offers ultra-high mass resolution capable of producing a similar dataset in a single run. However, for researchers who do not have access to this technology, the two-step approach allows for the detection and subsequent quantification of a relatively large number of chemicals without a priori knowledge of exposures.

## Supplementary Material

11356_2020_10702_MOESM1_ESM

11356_2020_10702_MOESM2_ESM

## Figures and Tables

**Table 1.** Demographic summary of study participants.

	Probands	Controls	Parents
*% Male*	83%	62%	47%
*Average Age (years)*	8.5	12.2	41.3
*Number of Subjects*	72	29	76

**Table 2.** Reported Concentrations from Targeted Method. LOD = Limit of Detection; Det. Rate = Percent of samples with results above the detection limit; <LOD = Value is below the limit of detection.

Name	LOD (ng/mL)	Total Det. Rate	Children (n=101)		Adults (n=76)	
			Det. Rate	Median [Maximum] (ng/mL)	Det. Rate	Median [Maximum] (ng/mL)
*1,2,3-Trichlorobenzene*	0.016	28%	30%	<LOD [0.219]	26%	<LOD [0.115]
*1,2,3,7,8-Pentachlorodibenzofuran*	0.028	5%	5%	<LOD [0.0588]	4%	<LOD [0.699]
*2,3,7,8-Tetrachlorodibenzofuran*	0.013	12%	11%	<LOD [0.0392]	14%	<LOD [0.346]
*2,4,6-Trichlorophenol*	0.015	82%	83%	0.285 [174]	80%	0.264 [1.75]
*Cyanazine*	5.0	0%	0%	<LOD [<LOD]	0%	<LOD [<LOD]
*Fluoranthene*	0.015	92%	94%	1.92 [30.0]	88%	1.62 [24.6]
*Fluorene*	0.48	78%	81%	5.44[26.8]	74%	3.29 [24.9]
*g-Chlordane*	0.064	17%	17%	<LOD [0.159]	17%	<LOD [0.172]
*Heptachlor*	0.029	10%	13%	<LOD [0.0633]	7%	<LOD [0.0705]
*Heptachlor Epoxide (Isomer B)*	0.15	1%	1%	<LOD [0.176]	1%	<LOD [0.211]
*Hexachlorobenzene*	0.025	66%	63%	0.0560 [0.292]	68%	0.0666 [0.283]
*Hexachlorobutadiene*	0.0079	65%	63%	0.401 [4.08]	67%	0.328 [189]
*Isosafrole*	0.029	76%	73%	0.297 [3.23]	80%	0261 [3.41]
*Metolachlor*	0.062	7%	6%	<LOD [0.278]	8%	<LOD [0.165]
Mirex*	0.019	30%	22%	<LOD [0.0974]	41%	<LOD [0.210]
*o,p′-DDE*	0.013	35%	36%	<LOD [0.0443]	34%	<LOD[0.0583]
*p,p’-DDT*	0.15%	3%	1%	<LOD [0.208]	5%	<LOD [0.645]
p,p′-DDE*	0.030	99%	99%	0.271 [118]	99%	0.568 [167]
*PBDE 28*	0.019	56%	52%	0.0196 [0.164]	62%	0.0233 [0.132]
*PBDE 100*	0.055	63%	63%	0.137 [6.06]	63%	0.129 [3.22]
*PBDE 154*	0.057	71%	71%	0.135 [2.01]	71%	0.140 [3.00]
*PCB 18*	0.023	51%	58%	0.0379 [0.431]	42%	<LOD [0.323]
*PCBs 28 and 31 summed*	0.023	84%	87%	0.164 [1.86]	80%	0.153 [1.62]
*PCB 66*	0.016	81%	82%	0.0619 [0.681]	79%	0.0468 [0.954]
*PCB 101*	0.024	58%	64%	0.0642 [1.80]	49%	<LOD [2.95]
*PCB 105*	0.018	54%	54%	0.0210 [0.582]	53%	0.0196 [0.449]
*PCB 118*	0.015	90%	90%	0.0807 [2.20]	89%	0.0827 [1.20]
*PCB 138*	0.047	65%	63%	0.0875 [3.11]	67%	0.106 [2.93]
*PCB 153*	0.035	76%	73%	0.107 [2.61]	80%	0.133 [1.38]
*PCB 156*	0.014	55%	50%	0.0141 [0.569]	61%	0.0188 [1.36]
*PCB 157*	0.018	7%	8%	<LOD [0.149]	7%	<LOD [151]
*PCB 167*	0.026	3%	4%	<LOD [0.133]	3%	<LOD [1.29]
*PCB 170*	0.046	53%	45%	<LOD [0.385]	64%	0.0585 [4.98]
PCB 180*	0.040	88%	84%	0.0821 [1.12]	93%	0.133 [5.68]
*PCB 187*	0.043	59%	54%	0.0496 [0.427]	66%	0.0568 [1.25]
PCB 194*	0.037	38%	26%	<LOD [0.152]	54%	0.0397 [4.15]
*PCB 206*	0.035	58%	57%	0.0406 [0.347]	59%	0.0468 [2.33]
*PCB 209*	0.066	53%	54%	0.0683 [0.531]	50%	<LOD [6.75]
*Pentachlorobenzene*	0.011	14%	17%	<LOD [6.00]	11%	<LOD [4.55]
Trans-nonachlor*	0.026	89%	85%	0.0635 [0.209]	93%	0.0868 [0.318]
*trans-Permethrin*	0.75	13%	13%	<LOD [1.85]	13%	<LOD [1.79]

* Significant difference between children and adults (rank-sum test, p-value < 0.002).

**Table 3.** Comparison of Selected Sample Medians to Age-Specific NHANES. NHANES results are the median whole-weight concentrations for individuals ages 30–50 years old in survey year 2003–04 (n=440–490, 47–48% male, varies by chemical). Adults in this study were 32–52 years old (n=78; 47% male). NHANES medians were computed using SAS Version 9.4 (Department of Health and Human Services: Centers for Disease Control and Prevention 2009).

Chemical	Adults in this Study (ng/mL)	NHANES Adults Ages 30–50 years (ng/mL)	Ratio of This Study to NHANES
*Hexachlorobenzene*	0.0635	0.090	
*p,p’-DDE*	0.564	1.24	0.5
*PCB 66*	0.0468	0.0081	5.8
*PCB 105*	0.0194	0.0064	3.0
*PCB 118*	0.0788	0.0307	2.6
*PCB 153*	0.133	0.127	1.0
*PCB 156*	0.0185	0.0201	0.9
*PCB 170*	0.0585	0.0395	1.5
*PCB 180*	0.130	0.1097	1.2
*PCB 187*	0.0568	0.0278	2.0
*PCB 194*	0.0397	0.0244	1.6
*PCB 206*	0.0455	0.0130	3.5
*Trans-nonachlor*	0.0864	0.0865	1.0

**Table 4.** Percent error for chemicals measured in NIST SRM 1958 using the method in this paper. Oxychlordane isomer, PBDEs 47, 99, 100, 153, and 183 were excluded because they had percent error >50% for more than one batch.

*Chemical Name*	*Certified Concentration (ng/kg)*	*Percent Error*				
		Batch 1	Batch 2	Batch 3	Batch 4	Batch 5
α-HCH	0.260	−8%	−6%	78%	−12%	5%
g-Chlordane	0.412	−35%	1%	−9%	−27%	15%
Hexachlorobenzene	0.442	−33%	−10%	−17%	−41%	20%
Mirex	0.384	−25%	7%	−1%	3%	−10%
o,p′-DDE	0.450	−14%	17%	−45%	22%	23%
Oxychlordane isomer	0.226	N/A	34%	−56%	51%	35%
p,p′-DDE	1.25	−29%	7%	−30%	11%	0%
p,p′-DDT	0.293	N/A^a^	N/A^a^	−64%	−26%	−28%
PBDE 28	0.462	−27%	−6%	−32%	−2%	−2%
PBDE 47	0.651	76%	163%	199%	233%	38%
PBDE 99	0.492	350%	301%	515%	248%	169%
PBDE 100	0.475	32%	47%	90%	29%	47%
PBDE 153	0.455	85%	N/A	−7%	52%	55%
PBDE 154	0.441	30%	N/A	9%	−45%	50%
PBDE 183	0.453	397%	4622%	N/A	28%	89%
PCB 18	0.407	−38%	−14%	2%	−23%	4%
PCB 66	0.414	−35%	−16%	−55%	13%	−9%
PCB 101	0.409	−37%	−25%	−23%	−8%	5%
PCB 105	0.419	−43%	−28%	−35%	−33%	−2%
PCB 118	0.412	−17%	−12%	−13%	−8%	6%
PCB 138	0.473	9%	21%	31%	2%	89%
PCB 153	0.457	−35%	−41%	−18%	−34%	1%
PCB 156	0.418	−36%	−20%	−34%	−30%	−2%
PCB 157	0.420	−39%	−15%	−34%	−30%	−8%
PCB 167	0.403	−45%	−26%	−36%	−41%	−15%
PCB 170	0.422	−40%	−10%	−16%	−17%	1%
PCB 180	0.459	−29%	−30%	−13%	−5%	6%
PCB 187	0.411	−37%	−25%	−18%	−25%	−6%
PCB 194	0.387	−27%	−14%	−6%	−26%	6%
PCB 195	0.385	−46%	−3%	168%	−32%	−7%
PCB 206	0.366	−22%	−11%	−7%	−19%	12%
PCB 209	0.338	−31%	−26%	26%	31%	36%
Trans-nonachlor	0.469	−34%	15%	−19%	−5%	−3%

a p,p’-DDT results for NIST SRM 1958 samples in Batches 1 and 2 were thrown out due to a coeluting peak which was observed only in the NIST SRM 1958 samples.
