# Supplementary material for: A Two-Step Gas Chromatography-Tandem Mass Spectrometry Method for Measurement of Multiple Environmental Pollutants in Human Plasma
Source: Environ Sci Pollut Res Int. Author manuscript; Available in PMC 2022 Jan 1. (PMC7790997; doi:10.1007/s11356-020-10702-6)
Supplement: 11356_2020_10702_MOESM1_ESM [file NIHMS1628007-supplement-11356_2020_10702_MOESM1_ESM.docx]

***Electronic Supplementary Information 1***

*Environmental Science and Pollution Research*

A Two-Step Gas Chromatography-Tandem Mass Spectrometry Method for Measurement of Multiple Environmental Pollutants in Human Plasma

Caitlin L. Johnson^a^, Elisa Jazan^a^, Sek Won Kong^b,c^*, and Kurt D. Pennell^d^*

^a^Department of Civil and Environmental Engineering, Tufts University, Medford, MA 02155, United States; ^b^Computational Health Informatics Program, Boston Children’s Hospital, Boston, MA 02115, United States; ^c^Department of Pediatrics, Harvard Medical School, Boston, MA 02115, United States; ^d^ School of Engineering, Brown University, Providence, RI 02912, United States

[*kurt_pennell@brown.edu](mailto:*kurt_pennell@brown.edu)

[*SekWon.Kong@childrens.harvard.edu](mailto:*SekWon.Kong@childrens.harvard.edu)

Online Resource 1: MRM Transitions and Collision Energies (CE, in volts) for all chemicals and two internal recovery surrogates measured in human plasma.

| **Chemical Class** | **CAS #** | **Name** |  | **Precursor Ion 1** | **Product Ion 1** | **CE 1** | **Precursor Ion 2** | **Product Ion 2** | **CE 2** | **Screening Method** | | **Used in Targeted Method?** |
| --- | --- | --- | --- | --- | --- | --- | --- | --- | --- | --- | --- | --- |
|  |  |  | **Abbreviation** |  |  |  |  |  |  | **Detection Rate** | **Analytical Quality** |  |
| **Polychlorinated dibenzo-p-dioxins and dibenzofurans (PCDDs/PCDFs)** | 38998-75-3 | 1,2,3,4,6,7,8-Heptachlorodibenzofuran |  | 407.8 | 345.0 | 36 | 409.8 | 347.0 | 36 | failed |  |  |
|  | 55684-94-1 | 1,2,3,4,7,8-Hexachlorodibenzofuran |  | 373.8 | 311.0 | 35 | 371.8 | 309.0 | 35 | failed |  |  |
|  | 57117-41-6 | 1,2,3,7,8-Pentachlorodibenzofuran |  | 339.8 | 277.0 | 35 | 337.8 | 275.0 | 35 |  |  | Yes |
|  | 51207-31-9 | 2,3,7,8-Tetrachlorodibenzofuran | 2,3,7,8-TCDF | 305.8 | 243.0 | 33 | 303.8 | 241.0 | 33 |  |  | Yes |
|  | 39001-02-0 | Octachlorodibenzofuran |  | 441.7 | 378.6 | 35 | 443.7 | 381.0 | 35 | failed |  |  |
|  | 35822-46-9 | 1,2,3,4,6,7,8-Heptachlorodibenzo-p-dioxin |  | 423.8 | 361.0 | 25 | 425.8 | 363.0 | 25 | failed |  |  |
|  | 39227-28-6 | 1,2,3,4,7,8-Hexachlorodibenzo-p-dioxin |  | 389.8 | 327.0 | 25 | 387.8 | 325.0 | 25 | failed |  |  |
|  | 40321-76-4 | 1,2,3,7,8-Pentachlorodibenzo-p-dioxin |  | 355.8 | 293.0 | 25 | 353.8 | 291.0 | 25 | failed |  |  |
|  | 1746-01-6 | 2,3,7,8-Tetrachlorodibenzo-p-dioxin | 2,3,7,8-TCDD | 321.8 | 259.0 | 24 | 319.8 | 257.0 | 24 | failed |  |  |
|  | 3268-87-9 | Octachlorodibenzo-p-dioxin |  | 459.8 | 396.8 | 26 | 457.8 | 394.8 | 26 | failed |  |  |
| **Flame Retardants, including Polybrominated Diphenyl Ethers (PBDEs)** | 183658-27-7 | 2-Ethylhexyl 2,3,4,5-tetrabromobenzoate |  | 420.4 | 392.6 | 0 | 111.4 | 55.0 | 0 | failed |  |  |
|  | 101-55-3 | 4-Bromodiphenyl ether | PBDE 3 | 248.0 | 141.0 | 20 | 248.0 | 220.1 | 10 | failed |  |  |
|  | 147217-78-5 | 2,4,4'-Tribromodiphenyl ether | PBDE 28 | 246.0 | 139.0 | 35 | 405.7 | 246.0 | 15 |  |  | Yes |
|  | 5436-43-1 | 2,2',4,4'-Tetrabromodiphenyl ether | PBDE 47 | 325.8 | 216.8 | 30 | 325.8 | 218.8 | 30 |  |  | Yes |
|  | 60348-60-9 | 2,2',4,4',5-Pentabromodiphenyl ether | PBDE 99 | 563.6 | 403.7 | 20 | 565.6 | 405.6 | 20 |  |  | Yes |
|  | 189084-64-8 | 2,2',4,4',6-Pentabromodiphenyl ether | PBDE 100 | 563.6 | 403.7 | 20 | 403.7 | 296.7 | 35 |  |  | Yes |
|  | 68631-49-2 | 2,2',4,4',5,5'-Hexabromodiphenyl ether | PBDE 153 | 483.7 | 323.6 | 40 | 643.6 | 483.6 | 20 |  |  | Yes |
|  | 207122-15-4 | 2,2',4,4',5,6'-Hexabromodiphenyl ether | PBDE 154 | 643.5 | 483.7 | 30 | 483.8 | 376.8 | 0 |  |  | Yes |
|  | 446255-26-1 | 2,2',3,4,4',5,5'-Heptabromodiphenyl ether | PBDE 180 | 561.7 | 454.7 | 40 | 721.8 | 561.7 | 30 | failed |  |  |
|  | 207122-16-5 | 2,2',3,4,4',5',6-Heptabromodiphenyl ether | PBDE 183 | 721.8 | 561.7 | 30 | 561.7 | 454.7 | 40 |  |  | Yes |
|  | 85-22-3 | Pentabromoethylbenzene |  | 499.2 | 484.7 | 0 | 484.5 | 405.7 | 0 | failed |  |  |
|  | 115-96-8 | tris(2-Chloroethyl) phosphate | TCEP | 143.0 | 80.8 | 0 | 143.0 | 116.6 | 0 |  |  | Yes |
|  | 13674-84-5 | tris(1-Chloro-2-propyl) phosphate | TCPP | 125.0 | 99.0 | 20 | 125.0 | 80.9 | 20 | N/A | failed |  |
| **Other Chemicals**  **Other Chemicals** | 87-61-6 | 1,2,3-Trichlorobenzene |  | 179.9 | 144.9 | 15 | 179.9 | 109.0 | 30 |  |  | Yes |
|  | 95-94-3 | 1,2,4,5-Tetrachlorobenzene |  | 214.0 | 178.9 | 20 | 216.0 | 180.9 | 20 |  |  | Yes |
|  | 120-82-1 | 1,2,4-Trichlorobenzene |  | 179.9 | 145.0 | 15 | 179.9 | 109.0 | 30 | N/A | failed |  |
|  | 95-50-1 | 1,2-Dichlorobenzene |  | 146.0 | 111.1 | 15 | 111.0 | 75.1 | 10 |  |  | Yes |
|  | 541-73-1 | 1,3-Dichlorobenzene |  | 146.0 | 111.0 | 15 | 146.0 | 75.0 | 30 |  |  | Yes |
|  | 106-46-7 | 1,4-Dichlorobenzene |  | 146.0 | 111.0 | 15 | 146.0 | 75.0 | 30 | N/A | failed |  |
|  | 134-32-7 | 1-Naphthylamine |  | 143.1 | 115.1 | 25 | 143.1 | 116.1 | 15 | N/A | failed |  |
|  | 58-90-2 | 2,3,4,6-Tetrachlorophenol |  | 230.0 | 130.9 | 30 | 130.9 | 96.0 | 20 | failed |  |  |
|  | 95-95-4 | 2,4,5-Trichlorophenol |  | 195.9 | 97.0 | 25 | 132.0 | 97.0 | 5 | N/A | failed |  |
|  | 88-06-2 | 2,4,6-Trichlorophenol |  | 131.9 | 97.0 | 10 | 196.0 | 97.0 | 30 |  |  | Yes |
|  | 120-83-2 | 2,4-Dichlorophenol |  | 162.0 | 63.0 | 30 | 98.0 | 63.0 | 5 | failed |  |  |
|  | 105-67-9 | 2,4-Dimethylphenol |  | 107.1 | 77.1 | 15 | 122.1 | 107.1 | 15 | N/A | failed |  |
|  | 121-14-2 | 2,4-Dinitrotoluene |  | 165.0 | 63.0 | 45 | 165.0 | 119.0 | 5 | failed |  |  |
|  | 606-20-2 | 2,6-Dinitrotoluene |  | 165.0 | 90.1 | 15 | 165.0 | 63.0 | 25 |  |  | Yes |
|  | 53-96-3 | 2-Acetamidofluorene |  | 181.1 | 180.1 | 15 | 223.1 | 181.1 | 15 | N/A | failed |  |
|  | 91-58-7 | 2-Chloronaphthalene |  | 162.0 | 127.1 | 20 | 127.0 | 77.0 | 15 | failed |  |  |
|  | 95-57-8 | 2-Chlorophenol |  | 128.0 | 64.0 | 15 | 128.0 | 63.0 | 30 |  |  | Yes |
|  | 91-57-6 | 2-Methylnaphthalene |  | 142.1 | 141.1 | 15 | 141.1 | 115.1 | 20 | N/A | failed |  |
|  | 95-48-7 | 2-Methylphenol |  | 107.0 | 77.0 | 15 | 108.0 | 107.0 | 15 | N/A | failed |  |
|  | 88-75-5 | 2-Nitrophenol |  | 138.9 | 109.0 | 5 | 138.9 | 81.0 | 15 | N/A | failed |  |
|  | 108-39-4 | 3-Methylphenol |  | 107.0 | 77.0 | 15 | 108.0 | 107.0 | 15 |  |  | Yes |
|  | 106-47-8 | 4-Chloroaniline |  | 127.0 | 65.0 | 20 | 127.0 | 92.0 | 15 | failed |  |  |
|  | 7005-72-3 | 4-Chlorophenyl phenyl ether |  | 141.1 | 115.1 | 20 | 204.0 | 141.1 | 20 | failed |  |  |
|  | 60-11-7 | 4-Dimethylaminoazobenzene |  | 225.1 | 120.1 | 10 | 120.1 | 77.0 | 20 | failed |  |  |
|  | 106-44-5 | 4-Methylphenol |  | 107.0 | 77.1 | 15 | 108.0 | 107.1 | 15 | N/A | failed |  |
|  | 56-57-5 | 4-Nitroquinoline-N-oxide |  | 128.0 | 101.0 | 10 | 174.0 | 116.0 | 10 | failed |  |  |
|  | 99-55-8 | 5-Nitro-o-toluidine |  | 152.0 | 106.0 | 10 | 106.0 | 77.0 | 15 | failed |  |  |
|  | 98-86-2 | Acetophenone |  | 105.0 | 77.0 | 15 | 77.0 | 51.0 | 15 | N/A | failed |  |
|  | 111-91-1 | bis(2-Chloroethoxy)methane |  | 93.0 | 63.0 | 5 | 95.0 | 65.0 | 5 |  |  | Yes |
|  | 111-44-4 | bis(2-Chloroethyl)ether |  | 93.1 | 63.0 | 0 | 95.1 | 65.0 | 5 |  |  | Yes |
|  | 132-64-9 | Dibenzofuran |  | 168.1 | 139.1 | 25 | 169.1 | 140.1 | 25 |  |  | Yes |
|  | 84-66-2 | Diethyl phthalate |  | 149.0 | 65.0 | 20 | 149.0 | 93.0 | 15 |  |  | Yes |
|  | 131-11-3 | Dimethyl phthalate |  | 163.0 | 77.0 | 20 | 163.0 | 135.0 | 10 | N/A | failed |  |
|  | 87-68-3 | Hexachlorobutadiene |  | 224.8 | 189.9 | 15 | 226.9 | 191.9 | 15 |  |  | Yes |
|  | 67-72-1 | Hexachloroethane |  | 200.9 | 165.9 | 15 | 202.9 | 167.9 | 15 |  |  | Yes |
|  | 1888-71-7 | Hexachloropropene |  | 212.9 | 118.9 | 20 | 212.9 | 116.9 | 20 | failed |  |  |
|  | 78-59-1 | Isophorone |  | 82.0 | 54.0 | 5 | 138.0 | 82.0 | 5 | N/A | failed |  |
|  | 120-58-1 | Isosafrole |  | 162.0 | 104.0 | 15 | 162.0 | 131.0 | 15 |  |  | Yes |
|  | 98-95-3 | Nitrobenzene |  | 77.0 | 51.0 | 15 | 123.0 | 77.0 | 10 | N/A | failed |  |
|  | 924-16-3 | N-Nitrosodi-n-butylamine |  | 84.1 | 56.0 | 15 | 116.0 | 99.1 | 0 |  |  | Yes |
|  | 621-64-7 | N-Nitrosodi-n-propylamine |  | 130.0 | 113.1 | 0 | 101.0 | 70.0 | 0 | failed |  |  |
|  | 95-53-4 | o-Toluidine |  | 107.1 | 106.1 | 15 | 106.1 | 77.0 | 20 | N/A | failed |  |
|  | 608-93-5 | Pentachlorobenzene |  | 249.9 | 215.0 | 20 | 248.0 | 213.0 | 20 |  |  | Yes |
|  | 87-86-5 | Pentachlorophenol |  | 265.9 | 167.0 | 25 | 265.9 | 165.0 | 25 | N/A | failed |  |
|  | 94-59-7 | Safrole |  | 162.1 | 104.1 | 15 | 131.1 | 103.1 | 10 | N/A | failed |  |
| **Polycyclic Aromatic Hydrocarbons (PAHs)**  **Polycyclic Aromatic Hydrocarbons (PAHs)** | 56-49-5 | 3-Methylcholanthrene |  | 268.1 | 252.1 | 35 | 268.1 | 253.1 | 15 | failed |  |  |
|  | 57-97-6 | 7,12-Dimethylbenz[a]anthracene |  | 256.1 | 241.1 | 15 | 241.1 | 239.1 | 25 | failed |  |  |
|  | 83-32-9 | Acenaphthene |  | 153.1 | 127.0 | 30 | 152.1 | 126.0 | 30 |  |  | Yes |
|  | 208-96-8 | Acenaphthylene |  | 152.1 | 126.0 | 30 | 152.1 | 102.1 | 30 |  |  | Yes |
|  | 120-12-7 | Anthracene |  | 178.1 | 152.1 | 25 | 178.1 | 151.1 | 30 | N/A | failed |  |
|  | 56-55-3 | Benz(a)anthracene |  | 228.1 | 226.1 | 30 | 114.0 | 101.1 | 10 | failed |  |  |
|  | 50-32-8 | Benzo(a)pyrene |  | 252.1 | 250.1 | 35 | 125.0 | 124.1 | 10 |  |  | Yes |
|  | 205-99-2 | Benzo(b)fluoranthene |  | 252.1 | 250.1 | 35 | 126.0 | 113.1 | 10 | N/A | failed |  |
|  | 207-08-9 | Benzo(k)fluoranthene |  | 252.1 | 250.1 | 30 | 126.1 | 113.1 | 10 | N/A | failed |  |
|  | 191-24-2 | Benzo[ghi]perylene |  | 138.0 | 137.0 | 15 | 137.0 | 136.0 | 15 | failed |  |  |
|  | 218-01-9 | Chrysene |  | 228.1 | 226.1 | 30 | 113.1 | 112.1 | 10 | N/A | failed |  |
|  | 53-70-3 | Dibenz[a,h]anthracene |  | 278.1 | 276.1 | 35 | 125.0 | 124.1 | 10 | N/A | failed |  |
|  | 206-44-0 | Fluoranthene |  | 201.1 | 200.1 | 15 | 202.1 | 152.1 | 30 |  |  | Yes |
|  | 86-73-7 | Fluorene |  | 166.1 | 165.1 | 15 | 165.1 | 164.1 | 20 |  |  | Yes |
|  | 193-39-5 | Indeno[1,2,3-cd]pyrene |  | 138.1 | 137.1 | 10 | 137.0 | 136.0 | 15 | failed |  |  |
|  | 91-20-3 | Naphthalene |  | 128.1 | 102.1 | 20 | 128.1 | 78.1 | 20 | N/A | failed |  |
|  | 85-01-8 | Phenanthrene |  | 178.1 | 152.1 | 25 | 176.1 | 150.1 | 25 |  |  | Yes |
|  | 129-00-0 | Pyrene |  | 201.1 | 200.0 | 15 | 200.1 | 174.0 | 25 |  |  | Yes |
| **Polychlorinated Biphenyls (PCBs)**  **Polychlorinated Biphenyls (PCBs)** | 37680-65-2 | 2,2',5-Trichlorobiphenyl | PCB 18 | 256.0 | 186.0 | 25 | 221.0 | 186.0 | 10 |  |  | Yes |
|  | 7012-37-5 | 2,4,4'-Trichlorobiphenyl^b^ | PCB 28^b^ | 256.0 | 186.0 | 25 | 258.0 | 186.0 | 25 |  |  | Yes |
|  | 16606-02-3 | 2,4',5-Trichlorobiphenyl^b^ | PCB 31^b^ | 256.0 | 186.0 | 25 | 258.0 | 186.0 | 25 |  |  | Yes |
|  | 41464-39-5 | 2,2',3,5'-Tetrachlorobiphenyl | PCB 44 | 255.0 | 220.0 | 10 | 289.9 | 219.9 | 30 | failed |  |  |
|  | 35693-99-3 | 2,2',5,5'-Tetrachlorobiphenyl | PCB 52 | 289.9 | 219.9 | 25 | 255.0 | 220.0 | 10 | failed |  |  |
|  | 33284-54-7 | 2,3,5,6-Tetrachlorobiphenyl^c^ | PCB 65^c^ | 289.9 | 219.9 | 20 | 291.9 | 219.9 | 20 |  |  | Yes |
|  | 32598-10-0 | 2,3',4,4'-Tetrachlorobiphenyl^a^ | PCB 66^a^ | 289.9 | 219.9 | 25 | 291.9 | 221.9 | 25 |  |  | Yes |
|  | 37680-73-2 | 2,2',4,5,5'-Pentachlorobiphenyl | PCB 101 | 325.9 | 255.9 | 30 | 253.9 | 184.0 | 35 |  |  | Yes |
|  | 32598-14-4 | 2,3,3',4,4'-Pentachlorobiphenyl ^a^ | PCB 105 ^a^ | 325.9 | 255.9 | 30 | 325.9 | 253.9 | 30 |  |  | Yes |
|  | 31508-00-6 | 2,3',4,4',5-Pentachlorobiphenyl | PCB 118 | 325.9 | 255.9 | 30 | 325.9 | 253.9 | 30 |  |  | Yes |
|  | 35065-28-2 | 2,2',3,4,4',5'-Hexachlorobiphenyl | PCB 138 | 359.9 | 289.9 | 30 | 287.9 | 217.9 | 40 |  |  | Yes |
|  | 35065-27-1 | 2,2',4,4',5,5’-Hexachlorobiphenyl | PCB 153 | 359.9 | 289.9 | 25 | 287.9 | 217.9 | 40 |  |  | Yes |
|  | 38380-08-4 | 2,3,3',4,4',5-Hexachlorobiphenyl ^a^ | PCB 156 ^a^ | 359.9 | 289.9 | 25 | 361.9 | 289.9 | 25 |  |  | Yes |
|  | 69782-90-7 | 2,3,3',4,4',5'-Hexachlorobiphenyl ^a^ | PCB 157 ^a^ | 359.9 | 289.9 | 30 | 361.9 | 289.9 | 30 |  |  | Yes |
|  | 41411-63-6 | 2,3,4,4',5,6-Hexachlorobiphenyl^c^ | PCB 166^c^ | 359.9 | 289.9 | 25 | 361.9 | 289.9 | 25 |  |  | Yes |
|  | 52663-72-6 | 2,3',4,4',5,5'-Hexachlorobiphenyl ^a^ | PCB 167 ^a^ | 359.9 | 289.9 | 25 | 361.9 | 289.9 | 25 |  |  | Yes |
|  | 35065-30-6 | 2,2',3,3',4,4',5-Heptachlorobiphenyl | PCB 170 | 393.8 | 323.8 | 30 | 393.8 | 358.8 | 15 |  |  | Yes |
|  | 35065-29-3 | 2,2',3,4,4',5,5'-Heptachlorobiphenyl | PCB 180 | 393.8 | 323.8 | 30 | 393.8 | 358.8 | 15 |  |  | Yes |
|  | 52663-68-0 | 2,2',3,4',5,5',6-Heptachlorobiphenyl ^a^ | PCB 187 ^a^ | 393.8 | 323.8 | 30 | 393.8 | 358.8 | 15 |  |  | Yes |
|  | 35694-08-7 | 2,2',3,3',4,4',5,5'-Octachlorobiphenyl | PCB 194 | 427.8 | 357.8 | 30 | 427.8 | 392.8 | 15 |  |  | Yes |
|  | 52663-78-2 | 2,2',3,3',4,4',5,6-Octachlorobiphenyl ^a^ | PCB 195 ^a^ | 427.8 | 392.8 | 15 | 429.8 | 394.8 | 15 |  |  | Yes |
|  | 40186-72-9 | 2,2',3,3',4,4',5,5',6-Nonachlorobiphenyl ^a^ | PCB 206 ^a^ | 461.8 | 391.7 | 30 | 463.8 | 393.7 | 30 |  |  | Yes |
|  | 2051-24-3 | Decachlorobiphenyl | PCB 209 | 497.7 | 427.7 | 30 | 495.8 | 425.7 | 30 |  |  | Yes |
| **Pesticides**  **Pesticides**  **Pesticides** | 96-12-8 | 1,2-Dibromo-3-chloropropane |  | 155.0 | 75.0 | 5 | 157.0 | 75.0 | 5 |  |  | Yes |
|  | 319-84-6 | α-Hexachlorocyclohexane | α-HCH | 216.9 | 181.0 | 5 | 218.9 | 183.0 | 5 |  |  | Yes |
|  | 5103-71-9 | a-Chlordane |  | 271.9 | 236.9 | 15 | 372.9 | 265.9 | 20 | failed |  |  |
|  | 15972-60-8 | Alachlor |  | 188.1 | 160.2 | 10 | 160.0 | 132.1 | 10 | failed |  |  |
|  | 309-00-2 | Aldrin |  | 262.9 | 192.9 | 35 | 254.9 | 220.0 | 20 | N/A | failed |  |
|  | 1912-24-9 | Atrazine |  | 214.9 | 58.1 | 10 | 214.9 | 200.2 | 5 | failed |  |  |
|  | 103-33-3 | Azobenzene |  | 77.0 | 51.0 | 15 | 105.0 | 77.1 | 5 | N/A | failed |  |
|  | 319-85-7 | β-Hexachlorocyclohexane | β-HCH | 181.0 | 145.0 | 15 | 216.9 | 181.1 | 5 | failed |  |  |
|  | 314-40-9 | Bromacil |  | 205.0 | 188.0 | 15 | 207.0 | 190.0 | 15 | failed |  |  |
|  | 510-15-6 | Chlorobenzilate |  | 139.1 | 111.0 | 10 | 251.1 | 139.1 | 15 | N/A | failed |  |
|  | 2675-77-6 | Chloroneb |  | 206.0 | 191.1 | 10 | 208.0 | 193.1 | 10 | N/A | failed |  |
|  | 54774-45-7 | cis-Permethrin |  | 183.1 | 168.1 | 10 | 183.1 | 165.1 | 10 | failed |  |  |
|  | 21725-46-2 | Cyanazine |  | 212.0 | 123.1 | 15 | 212.0 | 151.2 | 15 |  |  | Yes |
|  | 319-86-8 | δ-Hexachlorocyclohexane | δ-HCH | 217.0 | 181.1 | 5 | 181.1 | 145.1 | 15 | failed |  |  |
|  | 134-62-3 | N,N-diethyl-meta-toluamide | DEET | 119.1 | 91.0 | 10 | 119.1 | 65.1 | 20 |  |  | Yes |
|  | 333-41-5 | Diazinon |  | 137.1 | 84.0 | 10 | 137.1 | 54.0 | 20 | failed |  |  |
|  | 62-73-7 | Dichlorvos |  | 109.0 | 79.0 | 5 | 184.9 | 93.0 | 10 | N/A | failed |  |
|  | 60-57-1 | Dieldrin |  | 277.0 | 241.0 | 5 | 262.9 | 193.0 | 35 | failed |  |  |
|  | 122-39-4 | Diphenylamine |  | 169.0 | 168.2 | 15 | 168.0 | 167.2 | 15 | failed |  |  |
|  | 2921-88-2 | Dursban |  | 196.9 | 169.0 | 15 | 198.9 | 171.0 | 15 | N/A | failed |  |
|  | 944-22-9 | Dyfonate |  | 136.9 | 109.0 | 5 | 108.9 | 80.9 | 5 | failed |  |  |
|  | 959-98-8 | Endosulfan I |  | 194.9 | 159.0 | 5 | 194.9 | 160.0 | 5 | failed |  |  |
|  | 33213-65-9 | Endosulfan II |  | 206.9 | 172.0 | 15 | 194.9 | 158.9 | 10 | failed |  |  |
|  | 72-20-8 | Endrin |  | 262.8 | 193.0 | 35 | 244.8 | 173.0 | 30 | failed |  |  |
|  | 7421-93-4 | Endrin aldehyde |  | 249.9 | 214.9 | 30 | 278.9 | 209.0 | 25 | failed |  |  |
|  | 53494-70-5 | Endrin ketone |  | 316.8 | 100.8 | 10 | 316.8 | 280.7 | 5 | failed |  |  |
|  | 2593-15-9 | Etridiazole |  | 211.1 | 183.0 | 10 | 183.0 | 140.0 | 15 | failed |  |  |
|  | 58-89-9 | γ-Hexachlorocyclohexane | γ-HCH | 216.9 | 181.0 | 5 | 181.0 | 145.0 | 15 | failed |  |  |
|  | 5103-74-2 | g-Chlordane |  | 372.8 | 265.8 | 15 | 271.7 | 236.9 | 15 |  |  | Yes |
|  | 76-44-8 | Heptachlor |  | 271.7 | 236.9 | 15 | 273.7 | 238.9 | 15 |  |  | Yes |
|  | 28044-83-9 | Heptachlor epoxide (Isomer A) |  | 182.9 | 154.9 | 15 | 182.9 | 118.9 | 25 | failed |  |  |
|  | 1024-57-3 | Heptachlor epoxide (Isomer B) |  | 352.8 | 262.9 | 15 | 354.8 | 264.9 | 15 |  |  | Yes |
|  | 118-74-1 | Hexachlorobenzene |  | 283.8 | 213.9 | 30 | 283.8 | 248.8 | 15 |  |  | Yes |
|  | 51235-04-2 | Hexazinone |  | 171.0 | 71.1 | 10 | 171.0 | 85.1 | 10 | failed |  |  |
|  | 465-73-6 | Isodrin |  | 193.0 | 123.0 | 30 | 193.0 | 157.0 | 20 | failed |  |  |
|  | 121-75-5 | Malathion |  | 126.9 | 99.0 | 5 | 172.9 | 99.0 | 15 | N/A | failed |  |
|  | 950-37-8 | Methidathion |  | 144.9 | 85.0 | 5 | 144.9 | 58.1 | 15 | N/A | failed |  |
|  | 72-43-5 | Methoxychlor |  | 227.0 | 169.1 | 25 | 227.0 | 141.1 | 40 | failed |  |  |
|  | 298-00-0 | Methyl parathion |  | 262.9 | 109.0 | 10 | 125.0 | 47.0 | 10 | failed |  |  |
|  | 51218-45-2 | Metolachlor |  | 238.0 | 162.2 | 10 | 162.2 | 133.2 | 15 |  |  | Yes |
|  | 21087-64-9 | Metribuzin |  | 198.0 | 82.0 | 15 | 198.0 | 55.0 | 30 | failed |  |  |
|  | 2385-85-5 | Mirex |  | 271.8 | 236.8 | 15 | 273.8 | 238.8 | 15 |  |  | Yes |
|  | 53-19-0 | o,p'-Dichlorodiphenyldichloroethane | o,p’-DDD | 235.0 | 165.2 | 20 | 237.0 | 165.2 | 20 | failed |  |  |
|  | 3424-82-6 | o,p'- Dichlorodiphenyldichloroethylene | o,p’-DDE | 246.0 | 176.2 | 30 | 248.0 | 176.2 | 30 |  |  | Yes |
|  | 789-02-6 | o,p'- Dichlorodiphenyltrichloroethane | o,p’-DDT | 235.0 | 165.2 | 20 | 237.0 | 165.2 | 20 | failed |  |  |
|  | 27304-13-8 | Oxychlordane isomer |  | 114.9 | 51.1 | 25 | 114.9 | 87.0 | 15 |  |  | Yes |
|  | 72-54-8 | p,p'-Dichlorodiphenyldichloroethane | p,p’-DDD | 234.9 | 165.1 | 20 | 236.9 | 165.2 | 20 | failed |  |  |
|  | 72-55-9 | p,p'-Dichlorodiphenyldichloroethylene | p,p’-DDE | 246.1 | 176.2 | 30 | 315.8 | 246.0 | 15 |  |  | Yes |
|  | 50-29-3 | p,p'- Dichlorodiphenyltrichloroethane | p,p’-DDT | 235.0 | 165.2 | 20 | 237.0 | 165.2 | 20 |  |  | Yes |
|  | 56-38-2 | Parathion |  | 138.9 | 109.0 | 5 | 290.9 | 109.0 | 10 | N/A | failed |  |
|  | 82-68-8 | Pentachloronitrobenzene |  | 295.0 | 236.8 | 20 | 295.0 | 142.9 | 45 | failed |  |  |
|  | 2310-17-0 | Phosalone |  | 182.0 | 111.0 | 15 | 182.0 | 102.1 | 15 | N/A | failed |  |
|  | 29232-93-7 | Pirimiphos-methyl |  | 290.0 | 125.0 | 20 | 232.9 | 151.0 | 5 | failed |  |  |
|  | 122-34-9 | Simazine |  | 201.1 | 173.1 | 5 | 173.0 | 172.1 | 5 | failed |  |  |
|  | 39765-80-5 | Trans-nonachlor |  | 271.8 | 236.9 | 15 | 406.8 | 299.8 | 15 |  |  | Yes |
|  | 51877-74-8 | Trans-Permethrin |  | 183.1 | 168.1 | 10 | 183.1 | 165.1 | 10 |  |  | Yes |
|  | 1582-09-8 | Trifluralin |  | 305.9 | 264.0 | 5 | 264.0 | 160.1 | 15 | failed |  |  |

^a^chemical was added in Stage 2 of the method. ^b^Chemicals reported as a sum. ^c^Internal recovery surrogate.
