# Supplementary material for: A Two-Step Gas Chromatography-Tandem Mass Spectrometry Method for Measurement of Multiple Environmental Pollutants in Human Plasma
Source: Environ Sci Pollut Res Int. Author manuscript; Available in PMC 2022 Jan 1. (PMC7790997; doi:10.1007/s11356-020-10702-6)
Supplement: 11356_2020_10702_MOESM2_ESM [file NIHMS1628007-supplement-11356_2020_10702_MOESM2_ESM.docx]

***Electronic Supplementary Information 2***

*Environmental Science and Pollution Research*

A Two-Step Gas Chromatography-Tandem Mass Spectrometry Method for Measurement of Multiple Environmental Pollutants in Human Plasma

Caitlin L. Johnson^a^, Elisa Jazan^a^, Sek Won Kong^b,c^*, and Kurt D. Pennell^d^*

^a^Department of Civil and Environmental Engineering, Tufts University, Medford, MA 02155, United States; ^b^Computational Health Informatics Program, Boston Children’s Hospital, Boston, MA 02115, United States; ^c^Department of Pediatrics, Harvard Medical School, Boston, MA 02115, United States; ^d^ School of Engineering, Brown University, Providence, RI 02912, United States

[*kurt_pennell@brown.edu](mailto:*kurt_pennell@brown.edu)

[*SekWon.Kong@childrens.harvard.edu](mailto:*SekWon.Kong@childrens.harvard.edu)

Online Resource 2: Instrumental detection limits (IDL) for 31 chemicals. The IDL was calculated using 10 replicate injections of a low-concentration standard in hexane, and therefore does not account for matrix effects or variation introduced from the extraction method.

| **Analyte** | **IDL**  **(ng/mL in hexane)** |
| --- | --- |
| **α-HCH** | 0.08 |
| **β-HCH** | 0.05 |
| **δ-HCH** | 0.2 |
| **γ-HCH** | 0.05 |
| **o,p'-DDD** | 0.02 |
| **o,p'-DDE** | 0.02 |
| **o,p'-DDT** | 0.05 |
| **p,p'-DDD** | 0.07 |
| **p,p'-DDE** | 0.03 |
| **p,p'-DDT** | 0.06 |
| **PCB 77** | 0.005 |
| **PCB 81** | 0.01 |
| **PCB 101** | 0.003 |
| **PCB 105** | 0.004 |
| **PCB 114** | 0.03 |
| **PCB 118** | 0.03 |
| **PCB 123** | 0.01 |
| **PCB 128** | 0.005 |
| **PCB 138** | 0.004 |
| **PCB 153** | 0.001 |
| **PCB 156** | 0.003 |
| **PCB 157** | 0.01 |
| **PCB 167** | 0.003 |
| **PCB 169** | 0.02 |
| **PCB 170** | 0.01 |
| **PCB 180** | 0.005 |
| **PCB 187** | 0.004 |
| **PCB 189** | 0.02 |
| **PCB 195** | 0.01 |
| **PCB 206** | 0.005 |
| **PCB 209** | 0.004 |
